# Towards Clinical Translation of CD8^+^ Regulatory T Cells Restricted by Non-Classical Major Histocompatibility Complex Ib Molecules

**DOI:** 10.3390/ijms20194829

**Published:** 2019-09-28

**Authors:** Samiksha Wasnik, David J. Baylink, Jianmei Leavenworth, Chenfan Liu, Hongzheng Bi, Xiaolei Tang

**Affiliations:** 1 Department of Medicine, Division of Regenerative Medicine, Loma Linda University, Loma Linda, CA 92354, USA; 2 Department of Neurosurgery, the University of Alabama at Birmingham, Birmingham, AL 35294, USA; 3 Department of Microbiology, the University of Alabama at Birmingham, Birmingham, AL 35294, USA; 4 Department of Biomedical Sciences, College of Veterinary Medicine, Long Island University, Brookville, NY 11548, USA

**Keywords:** Qa-1, HLA-E, CD8^+^ Treg cells, non-classical major histocompatibility complex Ib molecules, epitopes, and vaccination

## Abstract

In central lymphoid tissues, mature lymphocytes are generated and pathogenic autoreactive lymphocytes are deleted. However, it is currently known that a significant number of potentially pathogenic autoreactive lymphocytes escape the deletion and populate peripheral lymphoid tissues. Therefore, peripheral mechanisms are present to prevent these potentially pathogenic autoreactive lymphocytes from harming one’s own tissues. One such mechanism is dictated by regulatory T (Treg) cells. So far, the most extensively studied Treg cells are CD4^+^Foxp3^+^ Treg cells. However, recent clinical trials for the treatment of immune-mediated diseases using CD4^+^ Foxp3^+^ Treg cells met with limited success. Accordingly, it is necessary to explore the potential importance of other Treg cells such as CD8^+^ Treg cells. In this regard, one extensively studied CD8^+^ Treg cell subset is Qa-1(HLA-E in human)-restricted CD8^+^ Treg cells, in which Qa-1(HLA-E) molecules belong to a group of non-classical major histocompatibility complex Ib molecules. This review will first summarize the evidence for the presence of Qa-1-restricted CD8^+^ Treg cells and their regulatory mechanisms. Major discussions will then focus on the potential clinical translation of Qa-1-restricted CD8^+^ Treg cells. At the end, we will briefly discuss the current status of human studies on HLA-E-restricted CD8^+^ Treg cells as well as potential future directions.

## 1. Introduction

Adaptive immune system is composed of central lymphoid tissues and peripheral lymphoid tissues. Human central lymphoid tissues mainly include thymus and bone marrow where lymphocytes develop. In the central lymphoid tissues, lymphocytes obtain the ability to react to foreign proteins (antigens) while those lymphocytes that are capable of reacting to self-proteins (autoreactive lymphocytes) and harming self-tissues are deleted. This process of deletion of harmful (pathogenic) autoreactive lymphocytes is called negative selection. Consequently, fully developed lymphocytes devoid of pathogenic autoreactive lymphocytes exit the central lymphoid tissues and populate peripheral lymphoid tissues such as spleen and lymph nodes. By virtue of the foregoing process, the adaptive immune system acquires the ability to spare one’s own tissues while attacking and eliminating any invaders.

However, the negative selection in central lymphoid tissues is not as perfect as originally thought. It is currently known that potentially pathogenic autoreactive lymphocytes are not completely eliminated in central lymphoid tissues and hence can be frequently found in peripheral lymphoid tissues [1,2]. Therefore, peripheral mechanisms must be in place to prevent these potentially pathogenic autoreactive lymphocytes from harming one′s own tissues. In this regard, multiple mechanisms have been described to keep the potentially pathogenic autoreactive lymphocytes at bay. One such mechanism is dictated by regulatory T (Treg) cells [3]. In this regard, the most extensively studied Treg cells are CD4^+^Foxp3^+^ Treg cells [4,5,6,7,8,9,10,11]. Due to strong preclinical results, CD4^+^Foxp3^+^ Treg cells are in the process of being translated into therapies for human diseases. Because the antigens that CD4^+^Foxp3^+^ Treg cells recognize remain largely unknown, most clinical trials use polyclonal CD4^+^Foxp3^+^ Treg cells whose antigen specificities are unknown [12,13,14,15,16,17]. So far, clinical trials using polycloncal CD4^+^Foxp3^+^ Treg cells for the treatment of immune-mediated diseases have met with limited success [12,13,14,15,16,17,18,19,20,21,22,23,24,25,26] (Table 1). For this reason, it has been suggested that exploration of other Treg cells, specifically CD8^+^ Treg cells, is warranted [3,27].

The necessity for further probing of CD8^+^ Treg cells is supported by recent findings that most CD8^+^ T cells infiltrating the tissues where autoimmune diseases occur were not pathogenic and actually played a regulatory role [3,27]. In this regard, CD8^+^ Treg cells were first described in 1970s [28]. However, when compared to CD4^+^Foxp3^+^ Treg cells, the study of CD8^+^ Treg cells significantly laps behind because of the difficulty in the specific characterization of such CD8^+^ Treg cells [29]. Despite the difficulty, more and more data now demonstrate that CD8^+^ Treg cells are an important arm of immune regulation [27,30,31,32,33,34,35,36]. Among various subsets of CD8^+^ Treg cells, CD8^+^ Treg cell subset, restricted by Qa-1(HLA-E in human) molecules, is the most extensively characterized CD8^+^ Treg cells because of its well-defined immune regulatory role [37,38,39,40]. Qa-1(HLA-E)-restricted CD8^+^ Treg cells are defined as those CD8^+^ Treg cells that use their T cell receptors (TCRs) to interact with Qa-1(HLA-E) molecules to mediate immune regulation. We believe that, based on the solid evidence on their immune regulatory role as described in the following sections, Qa-1(HLA-E)-restricted CD8^+^ Treg cells should be considered as potential therapies for human diseases.

A major characteristic of Qa-1(HLA-E)-restricted CD8^+^ Treg cells is that the TCRs on their cell surface can bind to Qa-1(HLA-E) molecules on the cell surface of target cells. In this regard, Qa-1(HLA-E) molecules belong to a group of non-classical major histocompatibility complex (MHC) Ib molecules. Although Qa-1(HLA-E) molecules are structurally similar to classical MHC Ia molecules, they are unique in several aspects. In one aspect, unlike classical MHC Ia molecules that are highly polymorphic, Qa-1(HLA-E) molecules have limited polymorphism. For example, only two functional HLA-E allotypes have been identified and they only differ by a single amino acid at position 107 (Arg for HLA-E*0101 and Gly for HLA-E*0103) [41]. In another aspect, under steady state, classical MHC Ia molecules are expressed at high levels on cell surface; in contrast, Qa-1(HLA-E) molecules are expressed at low levels. However, the expression levels of Qa-1(HLA-E) molecules on cell surface can be upregulated under certain conditions such as cell activation and stress [42,43]. We have proposed that these unique features endow Qa-1(HLA-E)-restricted CD8^+^ Treg cells with the ability to specifically regulate activated cells [37]. Further investigations into the importance of these unique features are justified.

**Figure 1:**
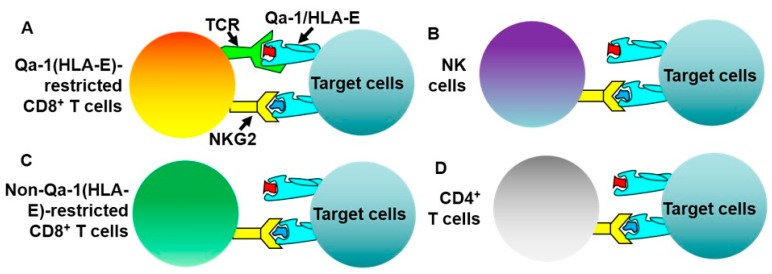
Qa-1(HLA-E)-restricted CD8^+^ T cells may express both TCRs and NKG2 receptors that are capable of interacting with Qa-1(HLA-E) molecules. Qa-1(HLA-E)-restricted CD8^+^ T cells (**A**) may express both TCRs and NKG2 receptors which can interact with Qa-1(HLA-E) molecules. NK cells (**B**), non-Qa-1(HLA-E)-restricted CD8^+^ T cells (**C**), and CD4^+^ T cells (**D**) only express NKG2 receptors. Color codes are used for distinguishing different types of cells and molecules only.

Although the major characteristic of Qa-1(HLA-E)-restricted CD8^+^ Treg cells is the interaction of their TCRs with Qa-1(HLA-E) molecules, Qa-1(HLA-E)-restricted CD8^+^ Treg cells may also express NKG2 receptors that can also interact with Qa-1(HLA-E) molecules (Figure 1). The differences between these two types of receptors are that Qa-1(HLA-E)-binding TCRs are specifically expressed on the cell surface of Qa-1(HLA-E)-restricted CD8^+^ Treg cells; however, NKG2 receptors can also be expressed on the cell surface of other cell subsets such as NK cells, non-Qa-1(HLA-E)-restricted CD8^+^ T cells, and CD4^+^ T cells [44,45]. In addition, NKG2 receptors include inhibitory and activating receptors [46,47,48]. Because of this dual presence of TCRs and NKG2 receptors on the cell surface of Qa-1(HLA-E)-restricted CD8^+^ Treg cells, regulatory functions, which were observed in CD8^+^ T cells and were thought to be the result of TCR-Qa-1(HLA-E) interaction in most previous studies, could be potentially due to NKG2-Qa-1(HLA-E) interaction. The foregoing confusion was recently cleared through the use of different Qa-1 mutant mouse strains (see detailed discussion in the following sections). These recent advances in the characterization of Qa-1-restricted CD8^+^ Treg cells have laid a solid foundation for the translation of Qa-1-restricted CD8^+^ Treg cells into therapies of human diseases.

Accordingly, this review will first summarize the evidence for the presence of Qa-1-restricted CD8^+^ Treg cells and their regulatory mechanisms. Major discussions will then be focused on the potential translation of Qa-1-restricted CD8^+^ Treg cells into therapies for human diseases. At the end, we will briefly discuss the current status of human studies on HLA-E-restricted CD8^+^ Treg cells as well as potential future directions.

## 2. Evidence That CD8^+^ T Cells Use TCRS to Interact with Qa-1/Peptide Complexes on the Cell Surface of Target Cells to Execute Immune Regulation

**Figure 2:**
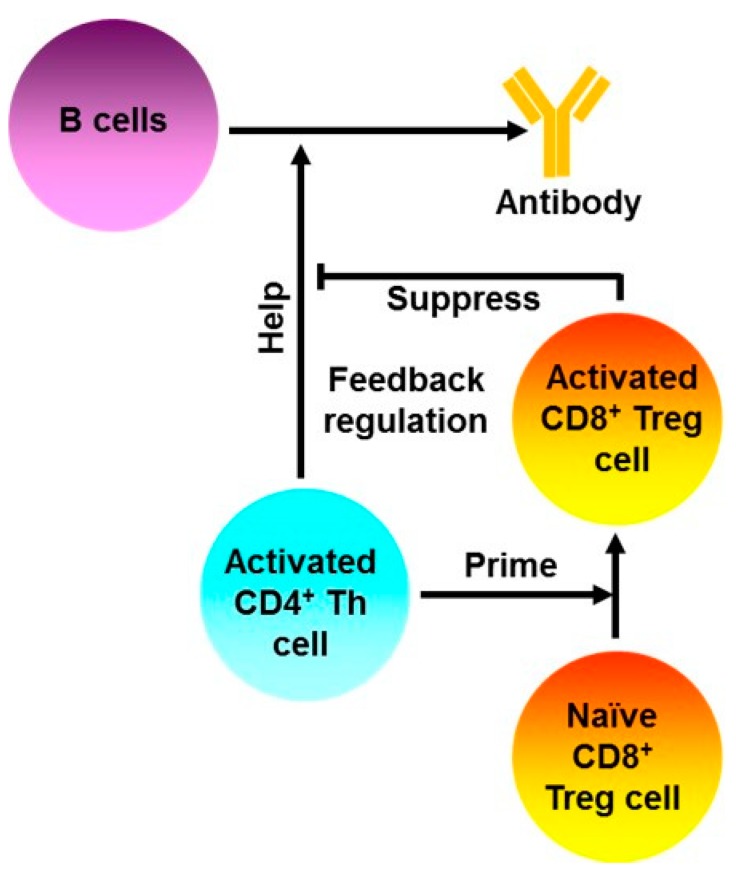
Activated CD4^+^ T cells prime CD8^+^ Treg cells. Activated CD4^+^ Th cells can activate a subset of CD8^+^ T cells that suppress the ability of the activated CD4^+^ Th cells to provide help for B cells to produce antibodies, a process called feedback regulation. Color codes are used for distinguishing different types of cells and molecules only.

In 1970s, it was shown that activated CD4^+^ T helper (Th) cells could prime CD8^+^ T cells. Such primed CD8^+^ T cells could subsequently suppress the ability of the activated CD4^+^ Th cells to provide help for B cells to produce antibodies (feedback regulation) (Figure 2) [49,50]. Interestingly, Qa-1^+^ but not Qa-1^-^ CD4^+^ Th cells could activate CD8^+^ T suppressors (or CD8^+^ Treg cells) [51]. These data suggest that Qa-1 molecules on the cell surface of activated CD4^+^ Th cells may be necessary for the activation of the CD8^+^ Treg cells. These early findings later led to more extensive investigation on CD8^+^ T cell-mediated, Qa-1-dependent regulation of activated CD4^+^ T cells in other experimental systems.

In one experimental system, mice were administered with staphylococcus enterotoxin B (SEB) that is a superantigen because it can bind specifically to TCRVβ8 protein and activate TCRVβ8^+^ T cells independent of antigen-specificity. Following the SEB administration, TCRVβ8^+^ CD4^+^ T cells in the mice were specifically activated and expanded to the maximal level on day 4. Subsequently, the number of TCRVβ8^+^ CD4^+^ T cells decreased to about 30%–40% below baseline levels [52,53,54,55]. Study of the SEB-administered mice showed that the numeric reduction of TCRVβ8^+^ CD4^+^ T cells was dependent on CD8^+^ T cells [34]. Further investigation demonstrated that CD8^+^ T cells from the SEB-administered mice showed TCRVβ8-specific cytotoxicity towards targets and the cytolytic activity was blocked by antisera to Qa-1, but not by antibody to classical MHC class Ia molecules. These data suggested that the CD8^+^ T cell-mediated killing of activated TCRVβ8^+^ cells depended on Qa-1 but not classical MHC Ia molecules. The concept of Qa-1-dependent immune regulation of activated CD4^+^ T cells was also supported by the findings in Qa-1-deficient mice in which exaggerated secondary CD4^+^ T cell responses to foreign and self-peptides were observed [33]. Accordingly, the foregoing data clearly demonstrate that CD8^+^ Treg cells can be de novo primed by activated CD4^+^ T cells and perform Qa-1-dependent feedback immune regulation.

Since Qa-1-restricted CD8^+^ T cells may express both Qa-1-binding TCRs and NKG2 receptors (Figure 1), the foregoing findings could not conclude which receptors were responsible for the Qa-1-dependent feedback regulation. This question was recently addressed using Qa-1 mutant mouse strains. In the mutant mice that were used to address this question, TCRs could not interact with Qa-1 molecules but NKG2 receptors still could. Studies of these mutant mice showed that, in the absence of TCR-Qa-1 interaction, immune responses to self-antigens [56,57], cancers [58], and viruses [59] were significantly augmented. These findings therefore definitively demonstrate that Qa-1-restricted CD8^+^ Treg cells are a bona fide Treg subset.

## 3. Potential Regulatory Mechanisms of Qa-1-Restricted CD8^+^ Treg Cells

An initial examination of the mechanisms by which Qa-1-restricted CD8^+^ Treg cells execute immune regulation was conducted in the aforementioned SEB-administered mice in 1990s [34]. It was found that CD8^+^ T cells from SEB-administered mice showed TCRVβ8-specific cytotoxicity towards activated CD4^+^ T cells and that the cytolytic activity could be blocked by antisera to Qa-1 molecules but not antibodies to classical MHC Ia molecules [34]. This ability of Qa-1-restricted CD8^+^ T cells to specifically kill target cells was also observed in Qa-1-restricted CD8^+^ T cell clones specific for a TCRVβ8.2-derived peptide (p42–50) [32,60]. Collectively, these data suggest that Qa-1-restricted CD8^+^ Treg cells are able to kill target cells in Qa-1-dependent manner in vitro.

In addition to the in vitro data mentioned above, in vivo experimental systems were also used to investigate the regulatory mechanisms of Qa-1-restricted CD8^+^ Treg cells. In an adoptive transfer system in which both CD8^+^ Treg cells and CD4^+^ T cells were administered into mice that do not have functional T, B, and NK cells, it was shown that the expressions of perforin and interferon-γ (IFN-γ) receptor but not IFNγand Fas ligand (FasL) in Qa-1-restricted CD8^+^ Treg cells were required for the suppression of activated CD4^+^ T cells [57]. In addition, studies of the protection from induction of experimental allergic encephalomyelitis (EAE) following p42-50 immunization demonstrated that regulatory functions of Qa-1-restricted p42-50-specific CD8^+^ Treg cells depended on perforin and IFN-γ but not Fas/FasL [61]. Moreover, in the study of regulation of T follicular helper (Tfh) cells, it was found that regulatory functions of Qa-1-restricted CD8^+^ Treg cells depended on perforin and interleukin-15 (IL-15) [56]. These data suggest that Qa-1-restricted CD8^+^ Treg cells require IFN-γ, IL-15, and perforin to effectively suppress the function of activated CD4^+^ T cells.

The foregoing in vitro and in vivo data appear to indicate that Qa-1-restricted CD8^+^ Treg cells regulate immune responses via their cytotoxic activity. Actually, cytotoxicity is the major mechanism that conventional CD8^+^ T cells use to eliminate targets such as cancer cells and virus-infected cells among others [62]. For other Treg cell subsets including CD4^+^Foxp3^+^ Treg cells, cytotoxicity is not the major regulatory mechanisms [63]. Therefore, some intriguing questions remain. The first question is whether Qa-1-restricted CD8^+^ Treg cells are simply a unique subset of effector CD8^+^ T cells. The second question is whether those regulatory mechanisms used by other Treg cells are also present in Qa-1-restricted CD8^+^ Treg cells. Answers to these issues should be forthcoming in the near future.

## 4. Harnessing Qa-1-Restricted CD8^+^ Treg Cells for the Treatment of Immune-Mediated Diseases

### 4.1. Studies on the Strategies that Can Enhance the Ability of Qa-1-Restricted CD8^+^ Treg Cells to Suppress Pathogenic Autoreactive CD4^+^ T Cells

Compelling evidence has now demonstrated that Qa-1-restricted CD8^+^ Treg cells can target activated lymphocytes to prevent exaggerated immune responses [33], which is important because uncontrolled activation of lymphocytes underlie some serious human diseases such as inflammatory bowel disease [64] and sepsis [65]. In addition, in patients with autoimmune diseases, pathogenic autoreactive lymphocytes are abnormally activated to attack self-tissues such as multiple sclerosis (MS) [66] and type 1 diabetes [67]. Accordingly, a question is whether the function of Qa-1-restricted CD8^+^ Treg cells can be intentionally augmented as a treatment strategy for the control of abnormal lymphocyte activation in patients with immune-mediated diseases. Below, we will review the data from these studies, discuss the challenges, and propose a potential solution.

#### 4.1.1. T Cell Vaccination Enhances the Feedback Regulatory Function of TCRVβ-Specific Qa-1-Restricted CD8^+^ Treg Cells in Vivo

Based on the findings that Qa-1-restricted CD8^+^ Treg cells are primed by activated CD4^+^ T cells during physiological immune responses [51,68], it was proposed that intentional vaccination with activated CD4^+^ T cells expressing a TCRVβ protein (T cell vaccination) should be able to enhance the function of Qa-1-restricted CD8^+^ Treg cells specific for the same TCRVβ protein. This concept was indeed supported by the finding that CD8^+^ T cells isolated from mice vaccinated with activated TCRVβ8^+^ CD4^+^ T cells showed Qa-1-dependent TCRVβ8-specific cytotoxicity towards target cells in vitro [69]. This ability of T cell vaccination to augment the function of TCRVβ-specific, Qa-1-dependent CD8^+^ Treg cells was later further confirmed in two other animal models of autoimmune disease [70]. one is herpes stromal keratitis which is a murine model of autoimmune disease following corneal infection by herpes simplex virus 1 and the other is non-obese diabetes which is a murine model of type 1 diabetes. These data suggest that vaccination with activated CD4^+^ T cells expressing a specific TCRVβ protein is a potential therapy for human diseases in which pathogenic autoreactive lymphocytes use the same TCRVβ protein.

#### 4.1.2. Vaccination with a TCRVβ8.2-Derived, Qa-1-Binding Peptide Prevents the Induction of EAE in Which Pathogenic CD4^+^ T Cells Predominantly Utilize TCRVβ8.2

Although T cell vaccination can suppress immune-mediated diseases through augmenting the function of TCRVβ-specific, Qa-1-restricted CD8^+^ Treg cells, T cells, in addition to Qa-1 epitopes, contain a myriad of other components that may activate unwanted immune responses. Therefore, it would be better to use defined Qa-1 epitopes derived from a TCRVβ protein as vaccines for the activation of TCRVβ-specific, Qa-1-restricted CD8^+^ Treg cells. To address this possibility, we identified a Qa-1 epitope (GLRLIHYSY or p42–50) [32,60] in the CDR1/2 region of TCRVβ8.2 protein. We showed that immunization with p42-50 prevented the induction of EAE in which pathogenic CD4^+^ T cells predominantly utilize TCRVβ8.2 protein [71,72,73]. In conclusion, our findings suggest that, to circumvent unnecessary immune suppression, peptide vaccination can replace T cell vaccination for augmenting the function of TCRVβ-specific, Qa-1-restricted CD8^+^ Treg cells.

#### 4.1.3. Immunization with Qa-1-Binding Peptides Derived from 60 KDa Heat Shock Protein (HSP60) Suppresses Pathogenic Autoreactive CD4^+^ T Cells in Several Autoimmune Disease Models

Although studies on the vaccination with Qa-1 epitopes derived from TCRVβ proteins are promising, we reason that this strategy is not practical during human applications. Firstly, although pathogenic CD4^+^ T cells in animal models of some autoimmune diseases may preferentially utilize certain TCRVβ proteins, the TCRVβ usage in pathogenic autoreactive CD4^+^ T cells in human patients is difficult to define. Secondly, epitopes recognized by pathogenic autoreactive T cells may evolve during the course of disease progression, a process called epitope spreading [74]. The epitope spreading makes TCRVβ usage within the population of pathogenic autoreactive T cells even more unforeseeable. Accordingly, TCRVβ-based strategy may be of limited clinical benefits.

To circumvent the limitation of TCRVβ-based strategies, it has been proposed that pathogenic autoreactive CD4^+^ T cells specifically present Qa-1 epitopes derived from HSP60. If this is the case, HSP60 Qa-1 epitopes, compared to TCRVβ Qa-1 epitopes, are better vaccines because they may potentially be applied to all pathogenic autoreactive lymphocytes. Studies of HSP60 have led to the discovery of two potentially functional Qa-1 epitopes in mouse HSP60 one is QMRPVSRAL (mHSP60sp) and the other is GMKFDRGYI (mHSP60p216). Data from one study showed that vaccination with dendritic cells (DCs) loaded with mHSP60sp significantly reduced the incidence and severity of paralytic disease in EAE mice [75]. It was proposed that the Qa-1/mHSP60sp complexes were preferentially expressed on the cell surface of pathogenic autoreactive CD4^+^ T cells in the mice induced for EAE [75]. Interestingly, it was also shown that CD8^+^ Treg cells appeared to block the development of spontaneous diabetes in non-obese diabetic mice by targeting the mHSP60sp on the cell surface of pathogenic autoreactive T cells [76]. On the other hand, in the study of mHSP60p216, it was shown that immunization with mHSP60p216-loaded DCs ameliorated clinical symptoms of collagen-induced arthritis (i.e., an animal model of human rheumatoid arthritis) but not of EAE [77]. Importantly, transfer of purified Qa-1/mHSP60p216 tetramer^+^ CD8^+^ T cells completely prevented the induction of collagen-induced arthritis [77]. Data from this study further suggest that Qa-1-restricted mHSP60p216-specific CD8^+^ Treg cells also target pathogenic autoreactive CD4^+^ T cells in vivo [77]. Collectively, the foregoing studies suggest that vaccination with HSP60 HLA-E epitopes can potentially be a promising therapy for autoimmune diseases.

#### 4.1.4. A Potential Novel Approach to Promote the Clinical Application of Qa-1-Restricted CD8^+^ Treg Cells for the Treatment of Autoimmune Diseases

With respect to the aforementioned HSP60-based strategy, there is so far no evidence that these HSP60 Qa-1 epitopes are indeed specifically expressed in pathogenic autoreactive lymphocytes in general. Because of the unpredictable nature of pathogenic autoreactive lymphocytes in a specific patient, we reason that it is highly unlikely that HSP60 Qa-1 epitopes are specifically and universally expressed in these cells. To support this reasoning, there is so far no evidence that corresponding human HSP60 HLA-E epitopes work for any human autoimmune diseases.

Considering the above-mentioned limitations facing the clinical translation of Qa-1 epitopes derived from pathogenic autoreactive CD4^+^ T cells, we recently asked whether Qa-1/HLA-E-restricted CD8^+^ Treg cells could suppress pathogenic autoreactive T cells by targeting Qa-1/HLA-E epitopes derived from tissue-specific antigens [78]. We have identified a Qa-1 epitope (MOG_196–204_ or MOG_196_) in murine myelin oligodendrocyte glycoprotein (MOG) which is a specific myelin protein in central nervous system (CNS). Using EAE induced by MOG_35–55_ in C57BL/6 mice, we showed that vaccination with MOG_196_-loaded DCs suppressed ongoing EAE. In addition, we showed that MOG_196_ vaccination activated CD8^+^ T cells that specifically accumulated in cervical lymph nodes in EAE-bearing mice, suggesting that the MOG_196_-specific Qa-1-restricted CD8^+^ Treg cells are specific for the demyelinating CNS. Moreover, the MOG_196_-mediated suppression of EAE depended on CD8^+^ T cells and the CD8^+^ T cells in vivo primed by MOG_196_-pulsed DCs transferred EAE suppression. These data further corroborate the concept that MOG_196_ vaccination indeed augments the function of CD8^+^ Treg cells. To support the potential translation into human applications, we have also proposed a strategy that is suitable for mapping Qa-1/HLA-E epitopes in a protein [79].

**Figure 3:**
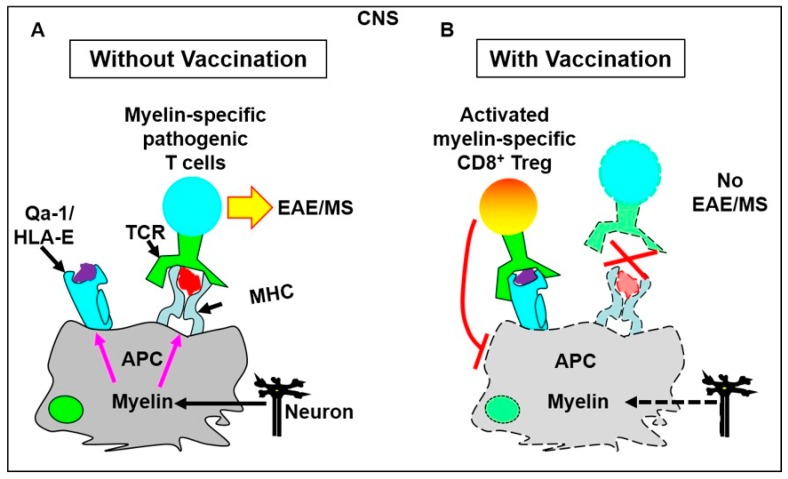
A novel therapeutic model of Qa-1/HLA-E-restricted CD8^+^ Treg cells. In the CNS of MS patients and EAE mice, antigen-presenting cells (APCs such as DCs) can phagocytose myelin proteins to present both pathogenic myelin epitopes and regulatory myelin Qa-1/HLA-E epitopes (e.g., MOG_196_). (**A**) In the absence of vaccination, Qa-1/HLA-E-restricted CD8^+^ Treg cells are not present in the CNS. Consequently, the APCs activate myelin-specific pathogenic T cells and perpetuate demyelinating disease (EAE/MS). (**B**) In contrast, vaccination with regulatory myelin Qa-1/HLA-E epitopes activates and expands Qa-1/HLA-E-restricted myelin-specific CD8^+^ Treg cells in peripheral lymphoid tissues. Such activated CD8^+^ Treg cells migrate into the CNS to target and inactivate the APCs. Consequently, the activation of myelin-specific pathogenic T cells is halted and the disease progression is stopped. Color codes are used for distinguishing different types of cells and molecules only.

Based on our findings, we now propose a novel therapeutic model of Qa-1/HLA-E-restricted CD8^+^ Treg cells using EAE/MS as an example (Figure 3). In this model, antigen-presenting cells (APCs such as DCs) in the CNS of MS patients can phagocytose myelin proteins to present myelin epitopes. Such myelin epitopes include pathogenic myelin epitopes and regulatory myelin Qa-1/HLA-E epitopes (e.g., MOG_196_). The pathogenic myelin epitopes can be recognized by myelin-specific pathogenic T cells and the regulatory Qa-1/HLA-E myelin epitopes can be recognized by Qa-1/HLA-E-restricted myelin-specific CD8^+^ Treg cells. In MS patients and EAE mice without vaccination, only myelin-specific pathogenic T cells and no activated Qa-1/HLA-E-restricted CD8^+^ Treg cells are present in the CNS. As a result, the APCs activate the myelin-specific pathogenic T cells to perpetuate demyelinating disease (EAE/MS). In contrast, vaccination with regulatory Qa-1/HLA-E myelin epitopes activates and expands Qa-1/HLA-E-restricted myelin-specific CD8^+^ Treg cells in peripheral lymphoid tissues. Such activated CD8^+^ Treg cells in peripheral lymphoid tissues then migrate specifically into the demyelinating CNS and can target the APCs that present both regulatory Qa-1/HLA-E myelin epitopes and pathogenic myelin epitopes. This targeting inactivates the APCs. Consequently, the activation of myelin-specific pathogenic T cells is halted and disease progression is stopped (Figure 3).

A caveat of Qa-1(HLA-E) epitope vaccination is that not all Qa-1(HLA-E) epitopes can induce CD8^+^ Treg cells. An example is the murine Qa-1 epitope Qdm (Qa-1-determinant modifier) that is part of the leader sequences of H-2D and L molecules (H-2D and L molecules belong to classical MHC Ia molecules). It has been shown that Qdm is the major epitope presented by Qa-1 molecules in resting cells and binds to inhibitory NKG2A receptor. In addition, we have also shown that Qdm is able to activate Qdm-specific CD8^+^ T cells in syngeneic hosts [77]. However, available data suggest that Qdm does not activate CD8^+^ Treg cells in vivo in several autoimmune disease models [76,77]. The foregoing findings suggest that different Qa-1 epitopes may activate functionally different CD8^+^ T cells. Accordingly, future studies are warranted to determine the nature of Qa-1 epitopes that is necessary for the activation of CD8^+^ Treg cells.

### 4.2. Studies on the Therapeutic Potentials of Qa-1-Restricted CD8^+^ T Cells for Cancers and Infections

Under some pathological conditions, e.g., cancers and infections, the functions of effector T cells are compromised. Hence, strategies are needed to enhance the functions of effector T cells. Therefore, it is of interest to know the roles of Qa-1-restricted CD8^+^ T cells in cancers and infections.

With respect to the role of Qa-1-restricted CD8 T cells in cancer pathogenesis, one study investigated the GVAX cancer vaccine (i.e., B16 melanoma cells engineered to overexpress granulocyte-macrophage colony-stimulating factor). This study showed that, in the absence of Qa-1-restricted CD8^+^ T cells, vaccination with the GVAX significantly suppressed the growth of B16 melanoma [58]. This observation supports that Qa-1-restricted CD8^+^ T cells play a regulatory role in the immune responses to cancers. In contrast, other studies suggest that Qa-1-restricted CD8^+^ T cells may act as effector cells in the immune response to cancers. In these studies, it was found that new Qa-1 epitopes were expressed in the cells that were deficient in antigen processing machineries such as transporter associated with antigen processing (TAP) [80] and endoplasmic reticulum aminopeptidase associated with antigen processing (ERAAP) [81]. Studies of animals deficient in TAP and ERAAP showed that Qa-1 epitopes expressed in these mice appeared to activate Qa-1-restricted cytotoxic CD8^+^ T cells. From the above findings, the authors proposed that Qa-1-restricted CD8^+^ T cells can kill the cells that are deficient in antigen processing such as cancer cells [80,81]. The reasons that led to the opposite conclusions regarding the role Qa-1-restricted CD8^+^ T cells in cancer pathogenesis from the foregoing previous findings are currently not clear. However, before Qa-1-restricted CD8^+^ T cells can be evaluated for the treatment of cancers, this dilemma will have to be resolved first.

With respect to the role of Qa-1-restricted CD8^+^ T cells in infections, in one study, it was shown that, in the absence of Qa-1-restricted CD8^+^ Treg cells, antiviral CD8^+^ T cell response to lymphocytic choriomeningitis virus was significantly enhanced during acute and chronic infections [59]. In addition, collateral tissue damage following lymphocytic choriomeningitis virus infection was significantly reduced, and the function of CD8^+^ effector T cells was significantly augmented [59]. These findings suggest that Qa-1-restricted CD8^+^ T cells play a regulatory role in the infection of lymphocytic choriomeningitis virus. However, another study that was performed in mice deficient in MHC class Ia molecules suggested otherwise [82]. Because of the deficiency in MHC class Ia molecules, CD8^+^ T cells in these mice are theoretically restricted by non-classical MHC Ib molecules including Qa-1 molecules. Studies of these mice showed that CD8^+^ T cells acted similarly to conventional CD8^+^ T cells (i.e., those CD8^+^ T cells that are restricted by MHC class Ia molecules), formed memory, and protected the mice from lethality during cytomegalovirus infection. Interestingly, Qa-1-restricted CD8^+^ T cells constituted a large and critical portion of the effector population [82]. Therefore, the previous findings on the paradoxical roles of Qa-1-restricted CD8^+^ T cells in infections are strikingly similar to those in cancers. Accordingly, before Qa-1-restricted CD8^+^ T cells can be evaluated for the treatment of cancers and infections, it is essential to fully understand the mechanisms underlying these paradoxical findings.

## 5. Current Status of Human Studies

Promising preclinical results on Qa-1-restricted CD8^+^ Treg cells have also spurred the investigation of potential benefits of HLA-E-restricted CD8^+^ Treg cells in humans. In this regard, genetic association studies have turned out encouraging. Firstly, it was shown that HLA-E gene polymorphism was associated with the development of several human diseases such as ankylosing spondylitis [83], Kawasaki disease [84], rheumatoid arthritis [85], type 1 diabetes mellitus [86], pemphigus vulgaris [87], psoriatic arthritis [88], and hepatitis C virus infection [89]. Secondly, it was also shown that tissue expression of HLA-E molecules was associated with the severity of liver disease during chronic hepatitis C infection [90] and with a worse survival of Tunisian patients with vulvar squamous cell carcinoma [91]. Data from these association studies suggest that HLA-E molecules are directly or indirectly involved in the pathogenesis of these serious diseases.

Mechanistically, HLA-E molecules may play this role by interacting with NKG2 receptors in various immune cell subsets (Figure 1), which is not a focus of this review. Alternatively, HLA-E molecules may play this role by interacting with TCRs in HLA-E-restricted CD8^+^ T cells (Figure 1). In this regard, potential HLA-E-restricted CD8^+^ T cells were observed in patients with several human autoimmune diseases such as MS [92,93] and type 1 diabetes [94]. These studies suggest that HLA-E-restricted CD8^+^ T cells play a regulatory role in patients with autoimmune diseases. For example, in one study, it was shown that freshly isolated CD8^+^ T cells from normal healthy control individuals consistently suppressed targets loaded with human HSP60sp (hHSP60sp; QMRPVSRVL); whereas this was not the case for the CD8^+^ T cells freshly isolated from type 1 diabetes patients [94]. Since hHSP60sp is a HLA-E-binding peptide [95], the data suggest that HLA-E-restricted hHSP60sp-specific CD8^+^ Treg cells in Type 1 diabetes patients are functionally impaired [94]. Interestingly, when CD8^+^ T cells from Type 1 diabetes patients were re-stimulated in vitro with hHSP60sp, the CD8^+^ T cells regained their suppressive activity. The data suggest that the functional impairment of HLA-E-restricted CD8^+^ Treg cells in type 1 diabetes can be corrected through in vitro re-stimulation [94]. However, it has not been established whether hHSP6sp is specifically expressed in the pathogenic autoreactive lymphocytes in type 1 diabetes patients. Neither has it been shown that hHSP60sp can be used for the treatment of any autoimmune diseases including type 1 diabetes patients. We reason that, because of the complexity of pathogenic autoreactive T cell repertoire in human patients with autoimmune diseases, the direct targeting of pathogenic autoreactive T cells may face formidable challenges. Therefore, tissue-specific regulatory HLA-E epitopes would seem to be more feasible therapy for human immune-mediated diseases.

In addition, putative HLA-E-restricted CD8^+^ T cells were also observed in patients who were infected with salmonella [96], hepatitis C virus [89], cytomegalovirus [97,98], and mycobacterium tuberculosis [99]. These studies appear to suggest that HLA-E-restricted CD8^+^ T cells act as effector cells rather than Treg cells in patients with infectious diseases. These findings are similar to some findings on Qa-1-restricted CD8^+^ T cells in animal infections [82]. However, studies of animal infections also suggest that Qa-1-restricted CD8^+^ T cells are Treg cells during infections [59]. Therefore, future clinical applications of HLA-E-restricted CD8^+^ T cells in infections and cancers will depend on whether the preclinical paradoxical findings can be fully understood.

## 6. Future Directions

Sufficient evidence has now been obtained to support that Qa-1-restricted CD8^+^ Treg cells are de novo activated during physiological immune responses and are an important regulatory arm in the peripheral immune system. In addition, previous data from other laboratories have demonstrated that Qa-1-restricted CD8^+^ Treg cells can be functionally augmented through vaccination to directly suppress pathogenic autoreactive T cells for the control of autoimmune diseases. However, the repertoire of pathogenic autoreactive T cells in human patients is unpredictable, which makes direct targeting of pathogenic autoreactive T cells in human patients difficult. For this reason, we have proposed a novel therapeutic model in which Qa-1-restricted CD8^+^ Treg cells may be harnessed to target tissue-specific antigens and thereby indirectly suppress pathogenic autoreactive T cells for the treatment of autoimmune diseases. We have presented initial evidence that support this novel therapeutic strategy using an animal model of MS. Further investigations are needed to determine whether this novel therapeutic model also works for other immune-mediated diseases such as type 1 diabetes, rheumatoid arthritis, and inflammatory bowel disease. In addition, answers are awaited as to whether tissue-specific regulatory HLA-E epitopes can be successfully mapped for the augmentation of tissue-specific HLA-E-restricted CD8^+^ Treg cells in humans and whether vaccination with tissue-specific regulatory HLA-E epitopes can be a novel therapy for human autoimmune diseases.

In addition, previous data have not conclusively demonstrated the exact roles of Qa-1(HLA-E)-restricted CD8^+^ T cells in cancers and infections. Future investigations are needed to clarify these roles so that HLA-E-restricted CD8^+^ T cells can be appropriately implemented for the treatment of these diseases.

## Figures and Tables

**Table 1:** Published clinical trials of Treg cells.

Diseases	Phase	# of Patients	Types of Treg Cells	Results	References
**GvHD**	I	2	In vitro expanded CD4^+^ CD25^+^CD127^−^ Treg cells	Safe. Chronic GvHD: significant symptom alleviation and reduced immune suppression for the longest time within all immunosuppressants used. Acute GvHD: transient improvement.	[18]
	I	23	In vitro expanded CD4^+^ CD25^+^ Treg cells	Safe but increased early opportunistic infections when Treg cells were present. Acute GvHD: Reduced incidence of grade II-IV.	[16,19]
	I	28	Freshly isolated CD4^+^ CD25^+^ Treg cells.	Safe. Reduced GvHD incidence. Enhanced immune reconstitution.Unaltered graft-versus-leukemia effect.	[15]
	II	43	Freshly isolated CD4^+^ CD25^+^ Treg cells.	Safe. Reduced GvHD incidence. Enhanced immune reconstitution. Reduced leukemia relapse.	[17]
	I	5	In vitro expanded CD4^+^ CD25^+^ Treg cells	Cancers found in 2 out of 5 patients. Improved chronic GvHD in 2 out of 5 patients. Stable chronic GvHD for 21 months in 3 out of 5 patients.	[20]
	I	12	IL-10-tolerized donor T cells	Safe. Four patients were disease- and immunosuppressant-free for at least 7.2 years after haplo-HSCT.	[21]
**Solid Organ Transplantation**	I	10	Donor-specifically tolerized lymphocytes.	Safe. Seven patients reached immunosuppressants-free for 16-33 months. Three patients developed mild rejection during weaning of immunosuppressants and resumed conventional immunosuppressants.	[22]
**Type 1 Diabetes**	I	10	In vitro expanded CD4^+^ CD25^+^CD127^−^ Treg cells.	Safe. 4–5 months after Treg cell infusion, eight patients still required <0.5 UI/Kg body wt of insulin daily. Two patients were completely insulin-free. 2 years after Treg cell infusion, the disease progressed and all patients were insulin-dependent.	[13,23,24,25]
	I	14	In vitro expanded CD4^+^ CD25^+^CD127^−^ Treg cells.	Safe.	[12,26]
**Refractory Crohn′s Disease**	I/II	20	In vitro cloned OVA-specific Tr1	Safe. 40% response rate based on a reduction in Crohn′s Disease Activity Index (CDAI).	[14]

## Abbreviations

APCs	Antigen-presenting cells.
CD4	Cluster of differentiation 4.
CD8	Cluster of differentiation 8.
CNS	Central nervous system.
DCs	Dendritic cells.
EAE	Experimental allergic encephalomyelitis.
ERAAP	Endoplasmic reticulum aminopeptidase associated with antigen processing.
Foxp3	Forkhead box P3.
GVAX	Tumor cells engineered to overexpress granulocyte-macrophage colony-stimulating factor.
hHSP60sp	Human HSP60 signal peptide (QMRPVSRVL).
HLA-E	Human leukocyte antigen-E. Human homologue of murine Qa-1.
HSP60	60 KDa heat shock protein.
IFN-γ	Interferon-γ.
IL-15	Interlecukin-15.
MHC	Major histocompatibility complex.
mHSP60sp	Murine HSP60 signal peptide (QMRPVSRAL).
mHSP60p216	Murine HSP60 peptide amino acid216-224 (GMKFDRGYI).
MOG	Myelin oligodendrocyte glycoprotein
MOG_196-204_	A Qa-1-binding peptide from murine myelin oligodendrocyte glycoprotein.
MS	Multiple sclerosis.
Qa-1	A group of murine non-classical major histocompatibility complex Ib molecules.
SEB	Staphylococcus enterotoxin B.
TAP	Transporter associated with antigen processing.
TCRs	T cell receptors.
TCRVβ8	T cell receptor β chain variable region 8.
Tfh cells	T follicular helper cells.
Th cells	T helper cells.
Treg cells	Regulatory T cells.
